# Periodicals Announced

**Published:** 1868-08-15

**Authors:** 


### *Periodicals Announced.*

*Half Year Compendium of Medical Science.*—The July (second) number of this valuable synopsis of professional literature is now in press. $3.00 *per annum.* Subscribers will address Dr. S. W. Butler, 115 South Seventh street, Philadelphia.

*Monthly Medical Reprint.*—John Hillyer, 14 South William street, New York, proposes the publication of a monthly reprint of articles from the English, French and German medical press. $5.00 *per annum.*

*London Lancet.*—The English publishers announce a colonial and foreign edition of their weekly issue in their paper, unabridged, containing even the local advertisements. $12.00 *per annum* (currency). Copyrighted articles by American writers are to be introduced. The object of this, of course, is to "kill off" the excellent and cheap (abridged) reprint by Wm. C. Herald, 32 Beekman street, New York, published at $5.00 *per annum.* Similar tactics were employed a number of years ago by the publishers of "Blackwood," and, if we remember rightly, succeeded in compelling the American republishers to divide the profits with them. We scarcely think that the foreign issue will achieve a success

*College Courant—Yale.*—Those of our friends wishing to keep up their acquaintance with college affairs—to revive old associations, and know of the whereabouts of old friends, will find about every thing necessary in this well conducted paper. Beyond tnis, it contains a large amount of entertaining and instructive reading, from some of the best writers in the country. It has recently been enlarged and improved. $4.00 *per annum.* Address—COLLEGE COURANT, New Haven, Conn.

